# Colonization dynamic and distribution of the endophytic fungus *Microdochium bolleyi* in plants measured by qPCR

**DOI:** 10.1371/journal.pone.0297633

**Published:** 2024-01-25

**Authors:** Pavel Matušinsky, Vendula Florová, Božena Sedláková, Patrik Mlčoch, Dominik Bleša

**Affiliations:** 1 Department of Botany, Faculty of Science, Palacký University in Olomouc, Olomouc, Czech Republic; 2 Department of Plant Pathology, Agrotest Fyto, Ltd, Kroměříž, Czech Republic; 3 Department of Experimental Biology, Faculty of Science, Masaryk University, Brno, Czech Republic; Universidade Federal de Minas Gerais, BRAZIL

## Abstract

*Microdochium bolleyi* is a fungal endophyte of cereals and grasses proposed as an ideal model organism for studying plant-endophyte interactions. A qPCR-based diagnostic assay was developed to detect *M*. *bolleyi* in wheat and *Brachypodium distachyon* tissues using the species-specific primers MbqITS derived from the *ITS* of the ribosomal gene. Specificity was tested against 20 fungal organisms associated with barley and wheat. Colonization dynamics, endophyte distribution in the plant, and potential of the seed transmission were analyzed in the wheat and model plant *B*. *distachyon*. The colonization of plants by endophyte starts from the germinating seed, where the seed coats are first strongly colonized, then the endophyte spreads to the adjacent parts, crown, roots near the crown, and basal parts of the stem. While in the lower distal parts of roots, the concentration of *M*. *bolleyi* DNA did not change significantly in successive samplings (30, 60, 90, 120, and 150 days after inoculation), there was a significant increase over time in the roots 1 cm under crown, crowns and stem bases. The endophyte reaches the higher parts of the base (2–4 cm above the crown) 90 days after sowing in wheat and 150 days in *B*. *distachyon*. The endophyte does not reach both host species’ leaves, peduncles, and ears. Regarding the potential for seed transmission, endophyte was not detected in harvested grains of plants with heavily colonized roots. Plants grown from seeds derived from parental plants heavily colonized by endophyte did not exhibit any presence of the endophyte, so transmission by seeds was not confirmed. The course of colonization dynamics and distribution in the plant was similar for both hosts tested, with two differences: the base of the wheat stem was colonized earlier, but *B*. *distachyon* was occupied more intensively and abundantly than wheat. Thus, the designed species-specific primers could detect and quantify the endophyte *in planta*.

## Introduction

Endophyte communities are found in almost all plants [1, 2]. These communities usually consist mainly of bacteria or fungi [3] that reside inside plant organs and do not damage the host tissue [3–7]. Some fungal endophytes can spend part of their life cycle as latent pathogens in plant tissues [8–10]. Fungal endophytes are vital in enhancing plant resilience against various stresses, such as abiotic factors like drought and biotic factors like pathogens and pests [11–13].

The presence of endophytes in plant tissues is typically determined using microscopic and media-cultivation methods [14–16]. However, DNA-based methods have become increasingly implemented [17]. PCR has been employed to detect endophytes in various grass tissues, including *Epichloë*, *Neotyphodium*, and *Acremonium* [18–21]. *Microdochium bolleyi* and *Microdochium phragmitis* were detected by nested PCR [22]. In addition to PCR, alternative methods such as PCR-DGEE (polymerase chain reaction and denaturing gradient gel electrophoresis) [23], Droplet Digital PCR Technology [24], microarray [25], sequencing [26], and immunological tests [27] can be employed to detect endophytes and other fungi residing in plant tissues. Furthermore, modern "omics" techniques such as comparative genomics, metagenomics, and metatranscriptomics have the potential to detect endophytes and unravel the relationships between plants and endophytes [28].

*Microdochium bolleyi* (*Mb*) is an endophytic fungus that primarily colonizes roots of cereals and grasses, including wheat [29], barley [30, 31], *Brachypodium distachyon* (*Bd*) [32], and others [33–35]. *Mb* has been observed to suppress various plant pathogens affecting cereals [29, 31, 36–39]. The formation of typical and under light microscopy visible chlamydospore clusters within colonized plant cells makes this endophytic fungus an excellent model organism to study plant endophyte interactions [32].

The first objective of this study was to develop species-specific primers for qPCR detection and quantification of endophyte *M*. *bolleyi* in plant tissues and thorough testing of their specificity and capability of detecting the endophyte in plants. The second aim of the study was to assess the colonization dynamic over time, the location of the endophyte in plants, and the seed transmission potential of the endophytic fungus *M*. *bolleyi* using wheat and the model plant species *B*. *distachyon*.

## Materials and methods

### Biological materials

All plants were cultivated in a cooled greenhouse (20/18°C, day/night) within pots 8 cm in diameter filled with a 50:50 planting substrate–sand (vol:vol) mixture, the substrate FLORCOM SV (BB Com, Letohrad, Czech Republic). For the experiment were used not treated seeds of the wheat cultivar Bohemia and of *B*. *distachyon* line Bd21 obtained from the Joint Genome Institute (https://jgi.doe.gov). Ten seeds were placed into each pot. After germination, the number of plants in every pot was reduced to six.

Six *Mb* isolates were used (UPOC-FUN-253–258) from the Collection of Phytopathogenic Microorganisms UPOC (Czech Republic). Inoculum of *Mb* isolates was obtained by cultivation on double sterilized millet seed [32], which was previously tested for the absence of *Mb* by qPCR. The inoculation of the plants was also carried out according to the method of Matušinsky et al. (2022) by adding inoculum to the seeds during the sowing [32]. Only sterile millet without endophyte was added to the control treatments.

Isolates of other fungi used for testing primers were obtained from four different collections of microorganisms (**Table 1**).

**Table 1:** Fungal species used in the analysis with the MbqITS primers designed in this study. A positive reaction result is indicated by a Cq lower than 30. The Cq levels in the table are the average of three replicates.

Species	Source	Code	Host	Cq average
*Microdochium bolleyi*	UPOC	UPOC-FUN-253	*Tritium aestivum*	11.25
*M*. *bolleyi*	UPOC	UPOC-FUN-254	*Tritium aestivum*	11.12
*M*. *bolleyi*	UPOC	UPOC-FUN-255	*Tritium aestivum*	11.29
*M*. *bolleyi*	UPOC	UPOC-FUN-256	*Tritium aestivum*	11.66
*M*. *bolleyi*	UPOC	UPOC-FUN-257	*Tritium aestivum*	11.05
*M*. *bolleyi*	UPOC	UPOC-FUN-258	*Tritium aestivum*	11.31
*Microdochium nivale*	AGT	13M30	*Tritium aestivum*	34.55
*Microdochium majus*	AGT	17M271	*Tritium aestivum*	34.52
*Cochliobolus sativus*	AGT	07CS4.3	*Hordeum vulgare*	37.54
*Ramularia collo-cygni*	AGT	20CZR19	*Hordeum vulgare*	36.69
*Oculimacula yallundae*	AGT	15OY119	*Tritium aestivum*	31.44
*Oculimacula acuformis*	AGT	15OA103	*Tritium aestivum*	32.28
*Rhizoctonia cerealis*	AGT	20CC88	*Tritium aestivum*	35.29
*Gaeumannomyces graminis* var. *tritici*	CCM	F-575	*Tritium aestivum*	>40
*Pyrenophora teres*	AGT	17PTT52	*Hordeum vulgare*	37.46
*Pyrenophora tritici-repentis*	AGT	19DTR6	*Tritium aestivum*	39.09
*Tilletia tritici*	AGT	06TCAR33	*Tritium aestivum*	36.95
*Fusarium graminearum*	AGT	20FG01	*Tritium aestivum*	36.48
*Fusarium culmorum*	AGT	19FcBd	*Brachypodium distachyon*	38.03
*Fusarium avenaceum*	CPPF	CPPF-161	*Tritium aestivum*	37.11
*Fusarium poae*	CPPF	CPPF-51	*Tritium aestivum*	35.62
*Fusarium langsethiae*	AGT	12FL4.00	*Avena sativa*	35.21
*Fusarium sporotrichioides*	CPPF	CPPF-146	*Tritium aestivum*	37.59
*Fusarium tricinctum*	CPPF	CPPF-254	*Tritium aestivum*	38.15
*Fusarium oxysporum*	AGT	19FOX06	*Zea mays*	37.26
*Zymoseptoria tritici*	AGT	ST-KM_B	*Tritium aestivum*	35.42

UPOC–Collection of Phytopathogenic Microorganisms, Czech Republic; AGT–Agrotest Fyto, Ltd, Czech Republic; CCM–Czech Collection of Microorganisms, Masaryk University, Faculty of Sciences, Czech Republic; CPPF–Collection of phytopathogenic fungi at Crop Research Institute Prague, Czech Republic.

### Sampling and assessment

To evaluate endophyte colonization by qPCR, plants were collected 30, 60, 90, 120, and 150 days after sowing/inoculation (dai), surface sterilized for 3 min in 1% NaOCl, and thoroughly rinsed in sterilized distilled water to remove all superficial hyphae and then dried. Plants were cut into individual parts: the lower part of the roots (distal part of the roots), roots 1 cm (part of the roots 1 cm below the crown), crown, base 1 cm (part of the stem base 1 cm above the crown), base 2–4 cm (part of the stem base 2–4 cm above the crown), peduncles, and ears (**S1 Fig**). To evaluate endophyte colonization by light microscopy, plants were collected in the same terms as above (30, 60, 90, 120, and 150 dai), and then fixed in 70% ethanol. Plants fixed in 70% ethanol were cleared in 2.5% KOH for three days, acidified in 1% HCl and stored in lactoglycerol [14]. Colonization of plant tissues by the endophytic fungus was assessed by microscopic examination (200× magnifications). Results were evaluated as positive when the visible presence of *Mb* chlamydospores was observed (**S2 Fig**). Estimation of root colonization level was made by an adapted method according to Trouvelot et al. (1986) [40].

Seeds of massively colonized plants were harvested and subsequently tested for *Mb* DNA by qPCR and sown to analyze the potential for endophyte transfer by seed. Plants grown from these seeds were tested for *Mb* chlamydospores by light microscopy and for *Mb* DNA content by qPCR.

### DNA isolation, primers design, and qPCR

Fungal mycelia (approximately 50–100 mg of biomass) of tested fungi (**Table 1**) were harvested from Petri dishes, ground to a fine powder in a cooled mortar using liquid nitrogen, then homogenized. Total genomic DNA was extracted using the DNeasy Plant Mini Kit (Qiagen, Germany). Plant material (approximately 50 mg of dried biomass per sample) was also ground to powder and extracted as described above. DNA concentration was measured using Qubit fluorometric quantification (ThermoFisher Scientific, Waltham, MA, USA), and DNA was diluted to a concentration of 5 ng μL^−1^.

Based upon sequences *ITS* (KP859018), *LSU* (large subunit of the ribosomal gene; KP858954), and *RPB2* gene (RNA 40 polymerase II second-largest subunit (KP859127)) [41] selected from the GenBank database, five primer pairs (MbqITSF/R, MbqLSU1F/R, MbqLSU2F/R, MbqPOL1F/R, and MbqPOL2F/R) were designed using Primer3Plus software [42]. The samples were analyzed using the CFX96TM Real-Time PCR Detection System (Bio-Rad, Hercules, CA, USA). The quantitative PCR mix consisted of 1× SYBR Green (Top-Bio, Vestec, Czech Republic), 0.2 μM forward and reverse primers (the best final primers MbqITSF/R were selected from a preliminary screen of the designed primers; (**S1 Table**)), 10 ng DNA (2 μL), and water to final volume 15 μL. The reference gene for wheat was *TaPAL* [43], and for *Bd*, it was *BdFIM* [44]. The control samples consisted of DNA of wheat or *Bd* plants colonized by the known level of root colonization by *Mb* obtained from the previous experiment; it will be further described in this article as a standard sample (sample of roots 1 cm under crown collected 90 days after sowing; wheat 20.6% and *Bd* with 23.4% of colonization measured by light microscopy method according to Trouvelot et al. (1986) [40]). The primers’ specificity and presence of primer dimers were verified by melting analysis. For the resulting MbqITSF/R primers, the reaction efficiency was analyzed by qPCR with DNA of *Mb* (isolate UPOC-FUN-253) and dilution series (1.0, 0.1, 0.01, 0.001, and 0.0001 ng).

### Test of primers specificity and diagnostic potential in plant tissues

The primers were tested on the six *Mb* isolates as described above. The designed primer pairs were tested for their specificity towards the DNA of fungi associated with diseases of wheat and other cereals. Fungal cultures were obtained from four microorganism collections (**Table 1**) and cultured on PDA. Furthermore, the potential for detection was tested in wheat, and *Bd* inoculated with the *Mb* compared with non-inoculated plants (inoculation and sampling as described above). All reactions were repeated three times.

### Statistical analysis

The data were analyzed using the 2^–ΔΔCq^ method with CFX Maestro software (Bio-Rad), and three biological and three technical replicates were run. The reaction efficiency was calculated using CFX Maestro software (Bio-Rad) from the slope value of the standard curve according to the formula E = (-1 + (10–1/slope) × 100) [45]. Differences in the relative quantity of the average values of inoculated samples were evaluated and compared to the standard sample (described above) by Tukey’s pairwise comparison test (*P* < 0.05; CFX Maestro software, Bio-Rad).

## Results

### Demonstrated root colonization by *Mb* of wheat and *Bd*

**Fig 1:**
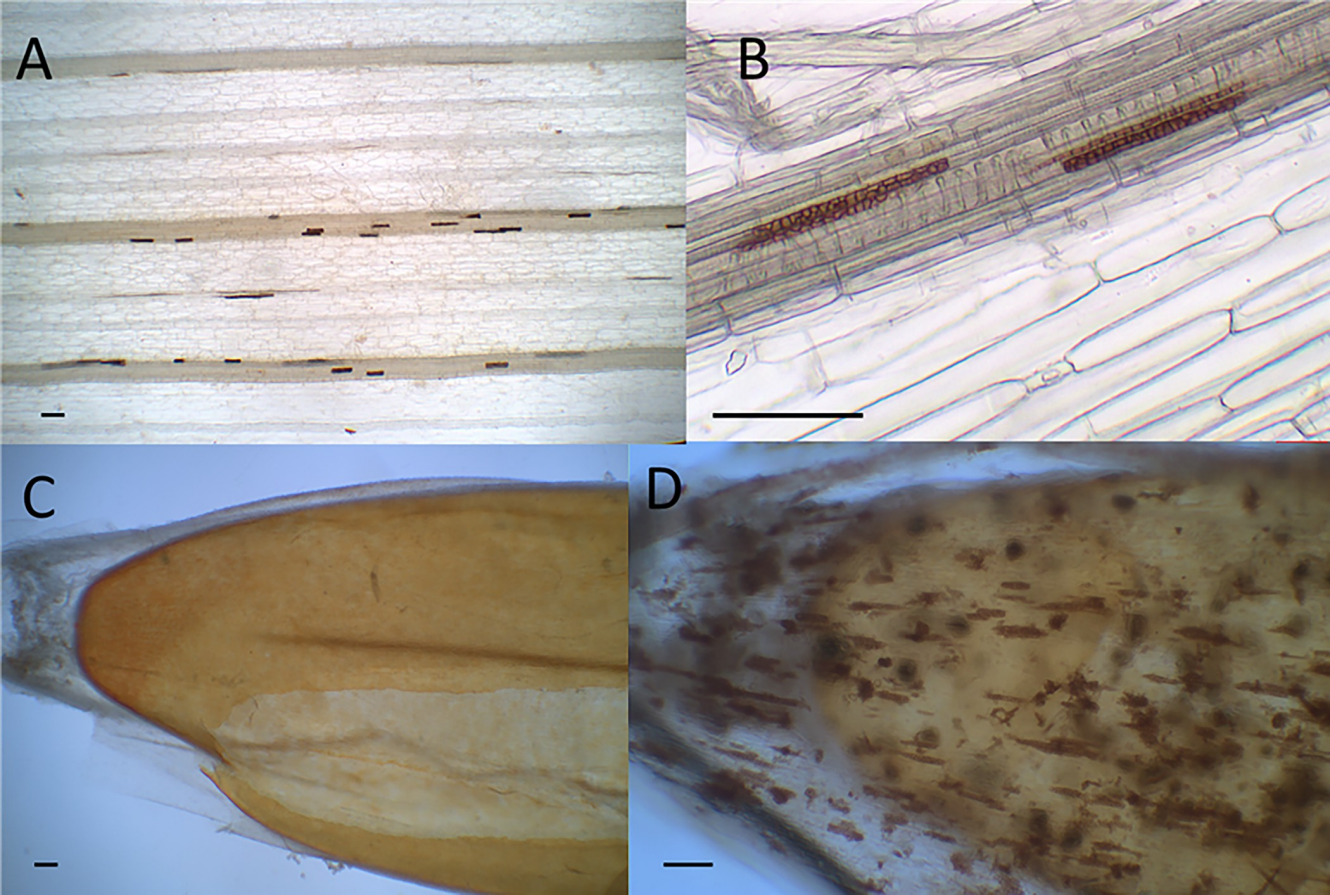
Chlamydospores of *Microdochium bolleyi* in wheat leaf sheaths (A, B) and comparison of seed coats of non-inoculated (C) and inoculated (D) *Brachypodium distachyon*. Scale bar = 100 μm.

Using microscopic and molecular methods, the presence of *Mb* was detected in both wheat and *Bd* samples. Using light microscopy, chlamydospores were observed in the seed coats, roots, crowns, and stem bases of wheat and *Bd* (**S2, S3 Tables and Fig 1**). No presence of chlamydospores was observed in plants without inoculation.

A set of MbqITSF/R best met the specificity requirements of the five designed primer pairs. The primers showed positive results in all six tested isolates of *Mb* (**S4 Table**). In the specificity test, the best-selected primers amplified DNA only from *Mb*, not any other fungal pathogens screened (**Table 1**). The primer set was therefore used to examine *Mb*-inoculated and non-inoculated plant material (wheat and *Bd*). Inoculated plants with chlamydospores present in plant tissues of both wheat and *Bd* showed positive results after qPCR with primers MbqITSF/R and, on the contrary, non-inoculated plants without chlamydospores in plant tissues showed negative results with Cq values higher than 30. The analysis confirmed that the primers were specific and robust in detecting and quantifying *Mb* within plant tissues. No false positive results were seen after melting analysis. Other tested primer pairs, MbqLSU1F/R, MbqLSU2F/R, MbqPOL1F/R, and MbqPOL2F/R, failed in the specificity test and were excluded from other parts of this study. The efficiency of the reaction was determined using a dilution series of DNA from the *Mb* isolate (UPOC-FUN-253). Based on the standard curve obtained, the efficiency (E) of the reaction was found to be 98.5% (**S5 Table**). The method showed a reliable positive response at 0.001 ng of *Mb* DNA (**S5 Table**), and the melting curve gives a single peak, indicating no artifacts.

### Dynamics of *Mb* colonization in wheat and *Bd* over time

**Fig 2:**
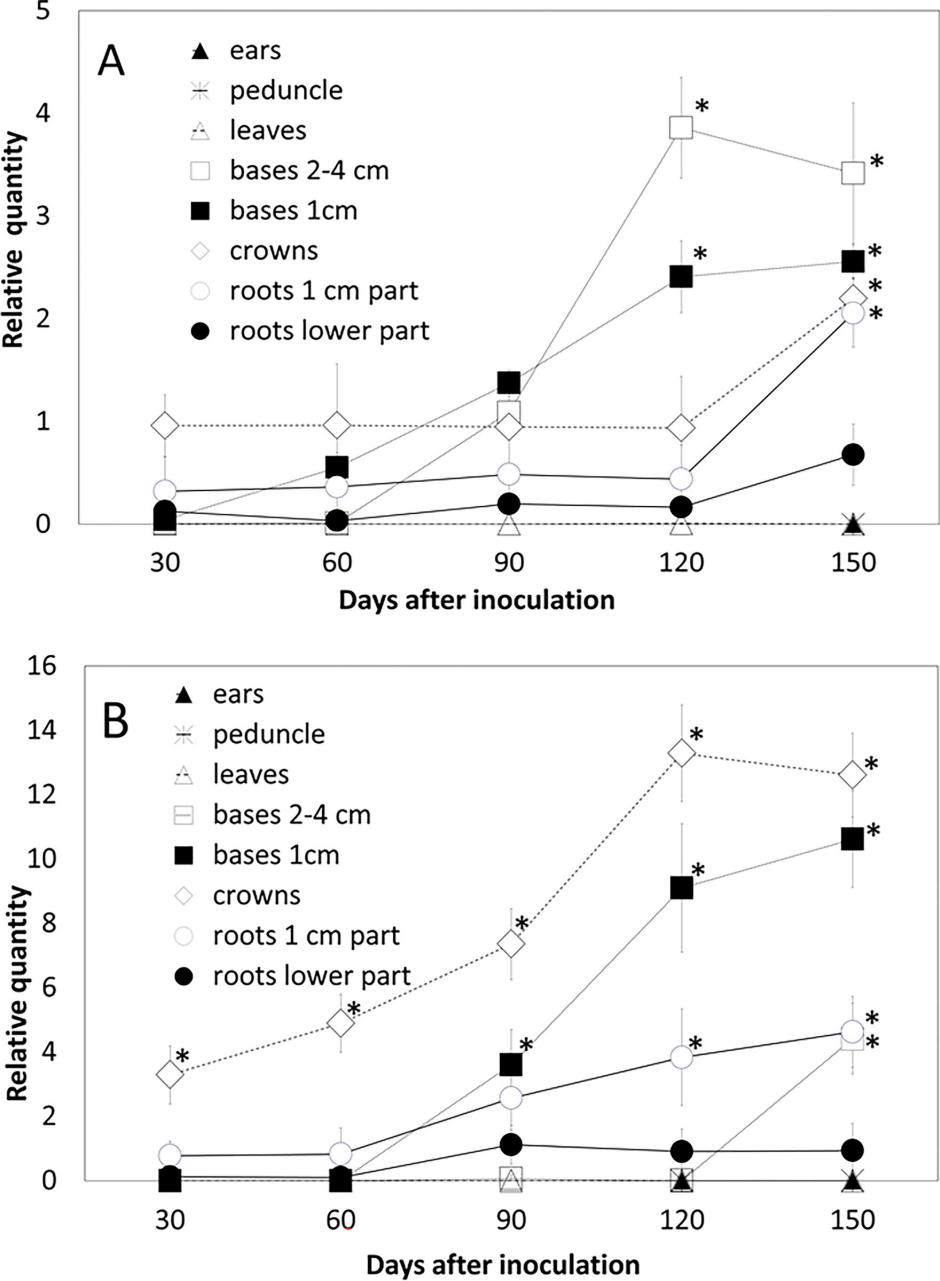
Dynamics of *Microdochium bolleyi* colonization in (A) wheat and (B) *Brachypodium distachyon* over time analyzed by qPCR. Statistically significant differences are indicated by asterixis (post hoc Tukey’s test, *P* < 0.05).

The seed coats, i.e., the already dead tissue, are massively colonized (30 days after inoculation) (**Fig 1C and 1D**). Only then are the living parts of the plant inhabited, such as the crown and roots, especially the part of the roots near the crown (30 dai). Over time (90 dai), the endophyte spreads to the distal parts of the roots and in wheat to the higher parts of the stem, 2–4 cm, but in *Bd*, only up to 1 cm and not higher. The DNA of the endophyte increases over time in the aerial parts, in wheat earlier than in *Bd* (**Fig 2**). In the underground parts, especially in the lower distal part of the roots, however, the level of endophyte DNA is growing very slowly during sampling periods (30–150 dai) with no significant changes (**Fig 2**). In the wheat above-ground parts, the presence of chlamydospores is confirmed microscopically in the outer sheath of the oldest leaf sheaths enclosing the stem (60–90 dai). Gradually, chlamydospores can be found in the sheaths of the second-oldest and third-oldest leaves (120–150 dai). Usually, the sheaths of leaves already in the senescence stage are colonized in this way, so a tendency for *Mb* to inhabit sheaths already in the senescence stage can be observed. On microscopy, it was found that most of the chlamydospores in the aerial part of the plants are in the cells adjacent to the vascular bundles (**Fig 1A and 1B**). Still, the fungus does not penetrate the vascular bundle itself, and the tissues are free of visual symptoms, i.e., without browning or other signs of damage. At the last sampling date of 150 days after sowing, the plants were already completely dry and dead, and the increase in *Mb* DNA in crown and roots 1cm above crown in wheat and stem base 2–4 cm above crow in *Bd* is, therefore, more likely due to the saprotrophic effect of the fungus. No *Mb* DNA was detected in leaf blades, peduncles, ears, and harvested seeds, nor was the presence of chlamydospores seen in these parts of the plants (**S2 and S3 Tables**).

### Distribution of *Mb* in wheat and *Bd* plants

The endophyte distribution in the plant varies over time, as indicated in the previous section of the results. Endophyte colonizes the roots, crowns, and stem bases of plants. The parts of the root adjacent to the crown are more intensively colonized than the distal parts of the roots, which are less colonized (**S2 and S3 Tables**). Crowns are inhabited initially, and the intensity is higher in the last phase of the plant’s life. The first tissues that are colonized after sowing are the seed coats (**Fig 1C and 1D**). Surprisingly, the plant’s above-ground parts, especially the lowest parts of the base, are inhabited significantly, and the intensity increases over time. The higher parts of the base, 2–4 cm, are also colonized in wheat. In contrast, in *Bd*, colonization in these higher regions of the base occurs only at the last sampling date, 150 days after sowing/inoculation. Neither leaf blades nor peduncles and ears are colonized even at the late stages of plant development (**S2 and S3 Tables**).

### Transmission by seed was not confirmed

Seeds from ten plants heavily colonized by the endophyte *Mb* were harvested and sown. These seeds and the plants grown from these seeds were screened for the presence of *Mb* by both light microscopy and qPCR. Neither the seeds nor any parts of the plants sown from these seeds contained endophyte *Mb* (**S6 Table**). Thus, it can be construed that *Mb* is not transmitted vertically, i.e., by seed.

## Discussion

The fungal endophyte of grasses and cereals *Mb* is a suitable model organism for studying endophyte interactions with plants. *Mb* can colonize several cereal species, including wheat [29], barley [30], and the model organism *Bd* [32]. Molecular methods for detecting *Mb* have previously been described by nested PCR [22] and standard PCR [32]. In the current study, newly developed primers for qPCR were designed and tested to quantify the amount of *Mb* DNA in host tissues. The colonization dynamics over time, the distribution of endophytes in different parts of the plant, and the potential of seed transfer, i.e., vertical transfer, were tested in wheat and *Bd*. Although microscopic methods have been used to determine the presence of endophytes, in many cases, they are combined with molecular or other methods [31, 46, 47]. However, light microscopy could be time-consuming and inaccurate compared to molecular techniques. Identifying individual organisms using microscopy requires considerable experience, as species can only be distinguished based on morphological characters in this way. In addition, individual roots are usually not evenly colonized along their entire length, so only a particular part of the roots needs to be selected for microscopy. If the roots are too thick, focusing the slide in all aspects is challenging. Microscopic detection should be used as a companion tool with a more reliable and accurate molecular method but not as a stand-alone method for diagnosing endophytes.

*Mb* is a partially saprotrophic organism; in some cases, slightly pathogenic effects on wheat have been observed [30, 48]. The presence of *Mb* has been previously described in roots [30], crowns [49], and stems of cereals [50]. We used newly developed primers to screen the dynamics of *Mb* colonization of plants. First, massive colonization of the seed coats occurs, and only then are colonized roots near the crown and parts of the stem base within 1 cm of the crown. Over time *Mb* spreads to the distal parts of the roots and, in wheat, to the higher parts of the stem. In the lower parts of the roots, the level of endophyte DNA is more or less the same at all sampling periods without significant changes. *Mb* content in roots part 1 cm bellow crown increases in the last sampling term, e.g., 150 dai in wheat and 120 and 150 dai in *Bd*. In above-ground parts, the presence of chlamydospores is confirmed microscopically in the outer sheath of the oldest leaf enveloping the stem. No *Mb* DNA was detected in leaf blades, peduncles, ears, and seeds, nor was the presence of chlamydospores in these plant parts. Seeds from plants heavily colonized by *Mb* endophyte were harvested and sown, and plants from these seeds were examined for the presence of *Mb* by both microscopic and qPCR methods. No *Mb* endophyte was detected in any parts of the plants sown from these seeds, and therefore, it can be concluded that *Mb* is not transmitted vertically, i.e., by seeds.

Our study is not the first to address the quantification of endophytes in plant tissues using qPCR. For example, Chow et al. (2018) [51] used qPCR to investigate the effect of endophytes (*Diaporthe phaseolorum*, *Trichoderma asperellum*, and *Penicillium citrinum*) on the pathogen *Ganoderma boninense* that infects oil palm (*Elaeis guineensis*). The analysis showed that when plants were inoculated simultaneously with endophytes, and the pathogen *G*. *boninense*, colonization by the pathogen was suppressed by endophytic fungi, as reflected by the higher DNA content of endophytes compared to that of the pathogen. Latz et al. (2021) [52] investigated interactions between plant genotypes and the biotic and abiotic environment affecting the plant microbiome using the *ITS1* metabarcoding method. Root microbial communities were less affected by abiotic factors than the phyllosphere microbiome. The diversity was highest in roots and lower in seeds, while leaves harbored the least diverse microbiome. The complete opposite was found in the work of Tao et al. (2008), who studied the distribution of fungal communities within leaf and root tissues of *Bletilla ochracea* (*Orchidaceae*) using random cloning, combined with DGGE (denaturing gradient gel electrophoresis) and phylogenetic analysis [53]. The main objective of their study was to compare the diversity of endophytes among roots and leaves of the same plants and to determine whether there is a consistency of communities within different organs of the same host. Fungal communities within leaf and root tissues differed significantly, and diversity within leaves was higher than within roots [53]. Bayman et al. (1997) used traditional isolation techniques and found that *Xylaria* spp. (*Xylariales*) and *Rhizoctonia*-like taxa (*Basidiomycota*) made up the majority of endophytes in epiphytic orchids, and fungal communities within leaves and roots were surprisingly similar [54].

These studies highlight the importance of conducting thorough investigations on endophytes and developing precise and reliable methods for detecting these organisms within the tissues of host plants. The use of molecular techniques in the determination of endophytes has wide applications. Cook et al. (2009) [55] used qPCR to determine the amount of an endophyte involved in producing a toxic alkaloid (swainsonine) in plants of the genus *Oxytropis* and *Astragalus*. The endophyte *Undifilum oxytropis*, involved in synthesizing the toxic alkaloid swainsonine, was quantified in plants of *Oxytropis sericea* and *Astragalus mollissimus* species using quantitative PCR. The amount of endophytes varies from one plant part to another and, in some cases, does not correspond to the concentration of swainsonine in the respective parts. The above works deal with microbial diversity inhabiting different parts of plants. Our work focused on one endophyte species and used qPCR to investigate the colonization dynamics and its distribution in the plant. The current study confirmed the endophyte presence in the roots and stem bases of wheat and *Bd*. *Mb* is found in roots, generally typical of the fourth class of endophytes to which this endophyte belongs [4]. Developing these newly designed primers has significantly broadened our understanding of the dynamics and distribution of endophytes within plants. Moreover, their application extends beyond our study, as they hold great potential for confirming successful colonization in commercial inoculations and facilitating further research in this field.

Fungal endophytes could be transmitted from plant to plant horizontally by soil or vertically by seed. A previous study by Sharon et al. (2023) [2] measured the correlation between fungal endophyte communities in seeds and stems of three cereal species under natural conditions and a controlled environment. Although most dominant fungal endophytic taxa could reach the seed internally, external seed infection (horizontal transfer) was found to be a primary source of specific taxa, including the most abundant species, *Alternaria infectoria*. Vertical transmission is not probably so frequent and is restricted to particular fungal taxa such as *Epichloë* [56]. Nevertheless, several studies indicate relatively more widespread vertical transfer than previously assumed [57, 58]. *Mb* belongs to the category of type 4 endophytes, and like other species in this group, it is recognized for its ability to be horizontally transmitted [4]. This has also been confirmed in our study. However, dead plants and debris remain on the ground and can be colonized by *Mb*. Murray and Gadd (1981) found that colonized seed remnants and roots remain in the ground after the crop is harvested and would provide a source of inoculum for the infection of barley seedlings in the following spring [30]. Regarding possible biological control, a potential approach could involve inoculation and colonization of seeds by *Mb* before sowing. By colonizing the seed coats, these cortical seed parts could serve as a source of inoculum and provide a favorable environment for seedlings’ inoculation. However, this hypothetical inoculation method has not yet been developed, and it would be helpful to test it in practice.

There are several reasons why the endophytic fungus *Mb* and plant *Bd* create an optimal model for studying plant-fungal interactions. The current study confirmed that the correspondence of *Mb* and *Bd* is excellent, and the fungus grows readily in *Bd* tissues. The inoculation method is simple, and the structures of *Mb* are readily visible in host tissues using light microscopy. In addition, we have developed and optimized species-specific primers for standard PCR [32] and here also for quantification of *Mb* by qPCR. The plant colonization dynamics by *Mb* are thoroughly studied in the current study. Previous studies have shown the beneficial effect of *Mb* on improving host plant immunity. Optimized methods and available primers from the last research [59, 60] allow the analysis of gene expression of specific marker genes in *Bd*. The advantage of *Mb* compared to strictly biotrophic symbiotic organisms is the possibility of cultivation on axenic media, and its massive sporulation ability makes its use suitable for large-scale production. Our results show that *Bd* and endophytic fungus *Mb* can be a model system for the plant-fungi interaction surveys. In general, a more profound knowledge of colonization dynamics and endophyte transmission could be used to improve agricultural management regarding plant growth promotion and biocontrol.

## Conclusion

*Mb* is a suitable model for studying the interactions of endophytic fungi and plants. Primers for qPCR have been developed for the diagnosis and relative quantification of endophyte *Mb* in plant tissues and tested in wheat and *Bd*. The method is suitable for confirming the presence of endophytes in plants, studying colonization dynamics and distribution in plants, and other applications.

## Supporting information

S1 Fig Schema of the sampling method. (TIF)

S2 Fig *Brachypodium distachyon* root with chlamydospores of *Microdochium bolleyi*. Scale bar = 100 μm.
(TIF)

S1 Table Primer pairs used in the study. Names, sequences of forward and reverse primers, publication sources of primer pairs, and gene functions are listed.
(DOCX)

S2 Table Colonization of wheat tissues by light microscopy and qPCR. (DOCX)

S3 Table Colonization of *Brachypodium distachyon* tissues by light microscopy and qPCR. (DOCX)

S4 Table Resulting Cq values after qPCR with five primer pairs. (DOCX)

S5 Table The efficiency (E) of the reaction was determined using a dilution series of DNA from the *Microdochium bolleyi* isolate (UPOC-FUN-253) with primers MbqITS. (DOCX)

S6 Table Seed transfer analysis by qPCR. (DOCX)
