# Supplementary figures and images for: Colonization dynamic and distribution of the endophytic fungus Microdochium bolleyi in plants measured by qPCR
Source: PLoS One. 2024 Jan 25;19(1):e0297633. doi: 10.1371/journal.pone.0297633 (PMC10810448; doi:10.1371/journal.pone.0297633)

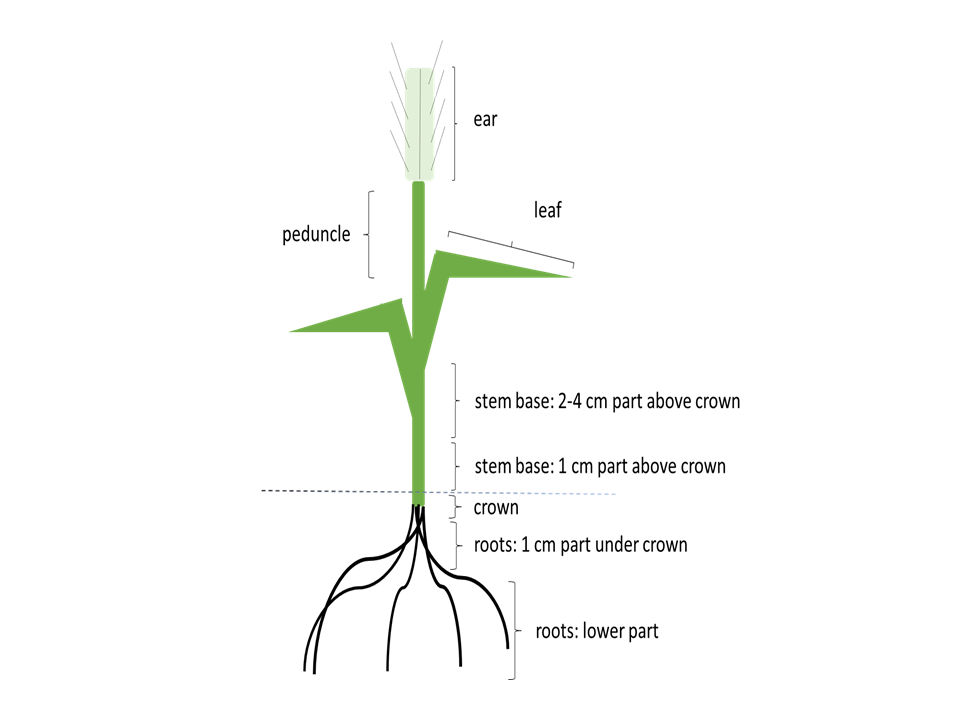

Supplement: S1 Fig — (TIF) [file pone.0297633.s001.tif]

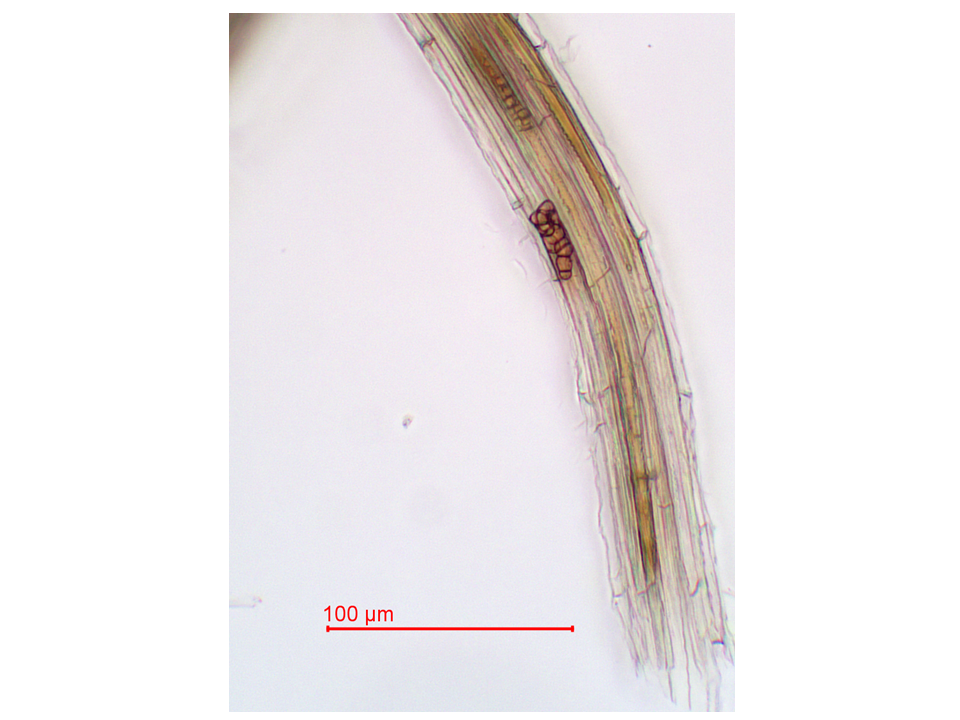

Supplement: S2 Fig — Scale bar = 100 μm. (TIF) [file pone.0297633.s002.tif]
