# Supplementary material for: Colonization dynamic and distribution of the endophytic fungus Microdochium bolleyi in plants measured by qPCR
Source: PLoS One. 2024 Jan 25;19(1):e0297633. doi: 10.1371/journal.pone.0297633 (PMC10810448; doi:10.1371/journal.pone.0297633)
Supplement: S1 Table — Names, sequences of forward and reverse primers, publication sources of primer pairs, and gene functions are listed. (DOCX) [file pone.0297633.s003.docx]

**Table S1. Primer pairs used in the study.** Names, sequences of forward and reverse primers, publication sources of primer pairs, and gene functions are listed.

| Gene name | Forward primer | Reverse primer | Publication | Function |
| --- | --- | --- | --- | --- |
| *TaPAL* | CGTTCTTGGTCGCGTTGTG | ACTCTTGACAGCATTCTTGACATTCT | 1 | Reference for *Triticum aestivum*  (phenylalanine ammonia-lyase gene) |
| *BdFIM* | CCTCACACGGATTTCGAGAGA | GGACAACCCATTTCTGCGA | 2 | Reference for *B. distachyon*  (fimbrin-like protein gene) |
| *MbqITS* | CGGTGCTGGAAACAGTGCTGCCA | CGATGCCAGAACCAAGAGATCC | Current study | ITS |

1. **Walsh, K., Korimbocus, J., Boonham, N., Jennings, P., Hims, M. (2005):** Using real-time PCR to discriminate and quantify the closely related wheat pathogens *Oculimacula yallundae* and *Oculimacula acuformis*. J. Phytopathol. 153: 715–721.

2. **Zhu, H., Wen, F., Li, P., Liu, X., Cao, J., Jiang, M., Feng, M., Chu, Z. (2014):** Validation of a reference gene (*BdFIM*) for quantifying transgene copy numbers in *Brachypodium distachyon* by real-time PCR. Applied biochemistry and biotechnology 172: 3163‒3175.
