# Supplementary material for: Colonization dynamic and distribution of the endophytic fungus Microdochium bolleyi in plants measured by qPCR
Source: PLoS One. 2024 Jan 25;19(1):e0297633. doi: 10.1371/journal.pone.0297633 (PMC10810448; doi:10.1371/journal.pone.0297633)
Supplement: S2 Table — (DOCX) [file pone.0297633.s004.docx]

**Tab S2 Colonization of wheat tissues by light microscopy and qPCR.**

| Plant part | Method | Days after inoculation | | | | |
| --- | --- | --- | --- | --- | --- | --- |
|  |  | 30 | 60 | 90 | 120 | 150 |
| Ears | Microscope | NO | NO | NO | NO | NO |
|  | qPCR | NEGATIVE | NEGATIVE | NEGATIVE | NEGATIVE | NEGATIVE |
| Peduncles | Microscope | NO | NO | NO | NO | NO |
|  | qPCR | NEGATIVE | NEGATIVE | NEGATIVE | NEGATIVE | NEGATIVE |
| Leaves | Microscope | NO | NO | NO | NO | NO |
|  | qPCR | NEGATIVE | NEGATIVE | NEGATIVE | NEGATIVE | NEGATIVE |
| Bases 2-4 cm | Microscope | NQ | NQ | NQ | NQ | NQ |
|  | qPCR | NEGATIVE | NEGATIVE | POSITIVE | POSITIVE | POSITIVE |
| Bases 1 cm | Microscope | NO | YES* | YES* | YES* | YES* |
|  | qPCR | NEGATIVE | POSITIVE | POSITIVE | POSITIVE | POSITIVE |
| Crowns | Microscope | YES (NQ) | YES (NQ) | YES (NQ) | YES (NQ) | YES (NQ) |
|  | qPCR | POSITIVE | POSITIVE | POSITIVE | POSITIVE | POSITIVE |
| Roots 1 cm | Microscope ** | YES (15.1%) | YES (15.6%) | YES (18.4%) | YES (18.9%) | YES (37.1%) |
|  | qPCR | POSITIVE | POSITIVE | POSITIVE | POSITIVE | POSITIVE |
| Roots lower part | Microscope ** | YES (0.7%) | YES (0.9%) | YES (5.2%) | YES (6.3%) | YES (13.6%) |
|  | qPCR | POSITIVE | POSITIVE | POSITIVE | POSITIVE | POSITIVE |

*Results of microscopy in the table described as YES – when chlamydospores are present and NO – when chlamydospores are not present, and qPCR with MbqITS primers as POSITIVE – when Cq values are lower than 30, and NEGATIVE – when Cq values are higher than 30.*

** Chlamydospores observed in leaf sheaths enveloped the base of the stem*

*** Percentage of colonization evaluated according to Trouvelot et al. (1986)*

*NQ Not quantified*

Trouvelot A, Kough JL & Gianinazzi-Pearson V (1986). Estimation of VA mycorhizal infection levels. Research for method having a functional significance. In : Physiological and Genetical Aspects of Mycorrhizae, V. Gianinazzi-Pearson and S. Gianinazzi (eds.). INRA Press, Paris, pp. 217-221. ISBN 2-85340-774-8.
