# Supplementary material for: Colonization dynamic and distribution of the endophytic fungus Microdochium bolleyi in plants measured by qPCR
Source: PLoS One. 2024 Jan 25;19(1):e0297633. doi: 10.1371/journal.pone.0297633 (PMC10810448; doi:10.1371/journal.pone.0297633)
Supplement: S3 Table — (DOCX) [file pone.0297633.s005.docx]

**Tab S3 Colonization of *B. distachyon* tissues by light microscopy and qPCR.**

| Organ | Method | Days after inoculation | | | | |
| --- | --- | --- | --- | --- | --- | --- |
|  |  | 30 | 60 | 90 | 120 | 150 |
| Ears | Microscope | NO | NO | NO | NO | NO |
|  | qPCR | NEGATIVE | NEGATIVE | NEGATIVE | NEGATIVE | NEGATIVE |
| Peduncles | Microscope | NO | NO | NO | NO | NO |
|  | qPCR | NEGATIVE | NEGATIVE | NEGATIVE | NEGATIVE | NEGATIVE |
| Leaves | Microscope | NO | NO | NO | NO | NO |
|  | qPCR | NEGATIVE | NEGATIVE | NEGATIVE | NEGATIVE | NEGATIVE |
| Bases 2-4 cm | Microscope | NQ | NQ | NQ | NQ | NQ |
|  | qPCR | NEGATIVE | NEGATIVE | NEGATIVE | NEGATIVE | POSITIVE |
| Bases 1 cm | Microscope | NQ | NQ | NQ | NQ | NQ |
|  | qPCR | NEGATIVE | NEGATIVE | POSITIVE | POSITIVE | POSITIVE |
| Crowns | Microscope | YES (NQ) | YES (NQ) | YES (NQ) | YES (NQ) | YES (NQ) |
|  | qPCR | POSITIVE | POSITIVE | POSITIVE | POSITIVE | POSITIVE |
| Roots 1 cm | Microscope * | YES (17.2%) | YES (19.3%) | YES (30.6%) | YES (35.4%) | YES (46.7%) |
|  | qPCR | POSITIVE | POSITIVE | POSITIVE | POSITIVE | POSITIVE |
| Roots lower part | Microscope * | YES (1.4%) | YES (1.6%) | YES (20.1%) | YES (19.6%) | YES (17.3%) |
|  | qPCR | POSITIVE | POSITIVE | POSITIVE | POSITIVE | POSITIVE |

*Results of microscopy in the table described as YES – when chlamydospores are present and NO – when chlamydospores are not present, and qPCR with MbqITS primers as POSITIVE – when Cq values are lower than 30, and NEGATIVE – when Cq values are higher than 30.*

** Percentage of colonization evaluated according to Trouvelot et al. (1986)*

*NQ Not quantified*

Trouvelot A, Kough JL & Gianinazzi-Pearson V (1986). Estimation of VA mycorhizal infection levels. Research for method having a functional significance. In: Physiological and Genetical Aspects of Mycorrhizae, V. Gianinazzi-Pearson and S. Gianinazzi (eds.). INRA Press, Paris, pp. 217-221. ISBN 2-85340-774-8.
