# Supplementary material for: Colonization dynamic and distribution of the endophytic fungus Microdochium bolleyi in plants measured by qPCR
Source: PLoS One. 2024 Jan 25;19(1):e0297633. doi: 10.1371/journal.pone.0297633 (PMC10810448; doi:10.1371/journal.pone.0297633)
Supplement: S4 Table — (DOCX) [file pone.0297633.s006.docx]

**Tab. S4 Resulting Cq values after qPCR with five primer pairs.**

| Species | Isolate | Cq (average from three replications) | | | | |
| --- | --- | --- | --- | --- | --- | --- |
|  |  | MbqITS | MbqLSU1 | MbqLSU2 | MbqPOL1 | MbqPOL2 |
| *M. bolleyi* | UPOC-FUN-253 | 11,75 | 13,83 | 13,09 | 18,22 | 19,18 |
| *M. bolleyi* | UPOC-FUN-254 | 11,38 | 13,87 | 12,53 | 18,01 | 18,93 |
| *M. bolleyi* | UPOC-FUN-255 | 11,00 | 13,70 | 13,00 | 17,80 | 18,88 |
| *M. bolleyi* | UPOC-FUN-256 | 11,39 | 13,76 | 13,21 | 17,78 | 19,11 |
| *M. bolleyi* | UPOC-FUN-257 | 11,52 | 13,77 | 13,12 | 17,99 | 19,20 |
| *M. bolleyi* | UPOC-FUN-258 | 11,29 | 13,84 | 13,29 | 17,86 | 18,98 |
| *M. nivale* | AGT-17M323 | 34,56 | 29,59 | 16,81 | 33,84 | >40 |
| *M. nivale* | AGT-13M205 | 34,17 | 29,48 | 16,99 | 33,73 | >40 |
| *M. majus* | AGT-13M195 | 34,37 | 29,38 | 17,16 | 33,62 | >40 |
| *M. majus* | AGT-14M71 | 34,25 | 29,52 | 17,01 | 33,76 | >40 |

*The lowest Cq values were achieved for Microdochium bolleyi using MbqITS primers. Parallel, these primers showed higher Cq values than 30 for the related fungal species Microdochium nivale and Microdochium majus.*
