# Supplementary material for: Colonization dynamic and distribution of the endophytic fungus Microdochium bolleyi in plants measured by qPCR
Source: PLoS One. 2024 Jan 25;19(1):e0297633. doi: 10.1371/journal.pone.0297633 (PMC10810448; doi:10.1371/journal.pone.0297633)
Supplement: S5 Table — (DOCX) [file pone.0297633.s007.docx]

**Tab S5** **The efficiency (E) of the reaction was determined using a dilution series of DNA from the *M. bolleyi* isolate (UPOC-FUN-253) with primers MbqITS.**

| DNA [ng] | Cq |
| --- | --- |
| 1 | 15,94 |
| 0,1 | 21,12 |
| 0,01 | 25,13 |
| 0,001 | 28,56 |
| 0,0001 | 29,01 |

*Based on the standard curve obtained, the efficiency of the reaction was found to be E = 98.5%.*
