# Supplementary material for: Colonization dynamic and distribution of the endophytic fungus Microdochium bolleyi in plants measured by qPCR
Source: PLoS One. 2024 Jan 25;19(1):e0297633. doi: 10.1371/journal.pone.0297633 (PMC10810448; doi:10.1371/journal.pone.0297633)
Supplement: S6 Table — (DOCX) [file pone.0297633.s008.docx]

**Tab S6 Seed transfer analysis by qPCR**

| Host species | Variant | Parental plants | | | Offspring | |
| --- | --- | --- | --- | --- | --- | --- |
|  |  | Plant code | Roots 1 cm under the crown | Seed | Plant code | Roots 1 cm under the crown |
| *Brachypodium distachyon* | Control variants of parental plants without endophyte *Microdochium bolleyi* | P1 | - | - | P1/O1 | - |
|  |  | P2 | - | - | P2/O2 | - |
|  |  | P3 | - | - | P3/O3 | - |
|  |  | P4 | - | - | P4/O4 | - |
|  |  | P5 | - | - | P5/O5 | - |
|  |  | P6 | - | - | P6/O6 | - |
|  |  | P7 | - | - | P7/O7 | - |
|  |  | P8 | - | - | P8/O8 | - |
|  |  | P9 | - | - | P9/O9 | - |
|  |  | P10 | - | - | P10/O10 | - |
|  | Parental plants inoculated by endophyte *Microdochium bolleyi* | P11 | + | - | P11/O11 | - |
|  |  | P12 | + | - | P12/O12 | - |
|  |  | P13 | + | - | P13/O13 | - |
|  |  | P14 | + | - | P14/O14 | - |
|  |  | P15 | + | - | P15/O15 | - |
|  |  | P16 | + | - | P16/O16 | - |
|  |  | P17 | + | - | P17/O17 | - |
|  |  | P18 | + | - | P18/O18 | - |
|  |  | P19 | + | - | P19/O19 | - |
|  |  | P20 | + | - | P20/O20 | - |
| Wheat | Control variants of parental plants without endophyte *Microdochium bolleyi* | P1 | - | - | P1/O1 | - |
|  |  | P2 | - | - | P2/O2 | - |
|  |  | P3 | - | - | P3/O3 | - |
|  |  | P4 | - | - | P4/O4 | - |
|  |  | P5 | - | - | P5/O5 | - |
|  |  | P6 | - | - | P6/O6 | - |
|  |  | P7 | - | - | P7/O7 | - |
|  |  | P8 | - | - | P8/O8 | - |
|  |  | P9 | - | - | P9/O9 | - |
|  |  | P10 | - | - | P10/O10 | - |
|  | Parental plants inoculated by endophyte *Microdochium bolleyi* | P11 | + | - | P11/O11 | - |
|  |  | P12 | + | - | P12/O12 | - |
|  |  | P13 | + | - | P13/O13 | - |
|  |  | P14 | + | - | P14/O14 | - |
|  |  | P15 | + | - | P15/O15 | - |
|  |  | P16 | + | - | P16/O16 | - |
|  |  | P17 | + | - | P17/O17 | - |
|  |  | P18 | + | - | P18/O18 | - |
|  |  | P19 | + | - | P19/O19 | - |
|  |  | P20 | + | - | P20/O20 | - |

*A + sign in the table indicates a positive reaction result distinguished by a Cq value lower than 30, and a – sign characterizes a negative response with a Cq value higher than 30. P parental plant, P/O offspring from the respective parental plant.*
